# An Interesting Case of Refractory Thrombotic Thrombocytopenic Purpura in the First Trimester of a Twin Pregnancy

**DOI:** 10.7759/cureus.47153

**Published:** 2023-10-16

**Authors:** Giuseppina Jacob, Amanpreet Dhaliwal, Vijay Chaudhary

**Affiliations:** 1 Internal Medicine, WakeMed Health and Hospitals, Raleigh, USA; 2 Hematology and Oncology, WakeMed Health and Hospitals, Raleigh, USA

**Keywords:** plasmapheresis, rituximab, caplacizumab, adamts13 deficiency, refractory ttp

## Abstract

Thrombotic thrombocytopenic purpura (TTP) is a rare pregnancy complication characterized by microangiopathic hemolytic anemia and consumption thrombocytopenia. We herein describe the case report of a 32-year-old woman who was six weeks pregnant with twins and developed thrombotic thrombocytic purpura (TTP). The patient had a history of sickle cell trait, migraines, and preeclampsia. She presented with complaints of nausea, fatigue, sore throat, and cough and was found to be anemic with a hemoglobin of 7 g/dl and thrombocytopenic with a platelet count of 8 x 10^3^/μL. The patient was promptly initiated on steroids and plasmapheresis with an excellent initial response. However, after three days, she developed a sudden onset headache and shortness of breath, and repeat labs showed worsening anemia (7.3 g/dl) and thrombocytopenia (8 x 10^3^/μL). ADAMTS13 activity was significantly low at 2%. Plasmapheresis was continued, and caplacizumab and rituximab treatment was initiated. The fetal ultrasound showed no cardiac activity in the fetal poles, and the patient had a dilation and curettage* *(D&C) for a missed abortion. She was discharged with a prednisone taper, daily caplacizumab, and weekly rituximab. This case report underscores the criticality of the prompt identification of TTP in its early stages, and appropriate management strategies for patients with refractory TTP (rTTP), including plasmapheresis, caplacizumab, and rituximab.

## Introduction

Thrombotic thrombocytic purpura (TTP) is a hematologic disorder that can manifest for the first time during pregnancy. Pregnancy itself is a prothrombotic state, which can complicate the diagnosis and management of conditions, such as TTP. Although TTP typically occurs in the third trimester, it can also occur in the first trimester, albeit rarely [1]. When TTP presents during pregnancy, it can lead to a challenging clinical scenario for both the mother and the developing fetus.

Clinical features are marked by the pentad of fever, microangiopathic hemolytic anemia, thrombocytopenia, and neurologic and renal abnormalities [2]. Many patients do not present with overt symptoms and are only diagnosed when lab works reveal decreased hemoglobin or platelet levels. Once the diagnosis of TTP is suspected, the initial standard of treatment is plasmapheresis exchange therapy. This therapy aims to replenish the patient's supply of ADAMTS13, a metalloprotease that cleaves large multimers of von Willebrand factor (vWF). When ADAMTS13 activity is reduced, large VWF multimers accumulate in the plasma. These large VWF multimers are more likely to bind to platelets and form aggregates. This can lead to the formation of microthrombi in small blood vessels throughout the body, which can cause organ damage and death. The addition of monoclonal antibodies, such as caplacizumab, may be required for refractory cases [2].

This case report documents the experience of a young woman, six weeks pregnant with twins, who developed TTP. The purpose of this report is to underscore the criticality of prompt identification of TTP in its early stages, outline appropriate management strategies for patients with refractory TTP (rTTP), and discuss the potential impact on the fetus. It also underscores the potential for favorable outcomes, even in challenging cases, with appropriate treatment strategies.

## Case presentation

A 32-year-old G2P0101 woman with a medical history significant for sickle cell trait and postpartum pre-eclampsia (resolved with nifedepine and magnesium sulfate) presented to an urgent care center with complaints of nausea, fatigue, sore throat, and cough for 10 days. Her vital signs were stable, but initial labs showed the patient to be anemic, with a hemoglobin level of 7 g/dL, a hematocrit level of 20%, and a mean corpuscular volume (MCV) of 76 fL, and profoundly thrombocytopenic with a platelet count of 8 x 10^3/μL (Table 1), a urinalysis positive for protein and hemoglobin, and a positive pregnancy test. She was transferred to our emergency department for further workup. 

**Table 1:** Complete blood cell panel labs WBC, white blood cells; RBC, red blood cells; MCV, mean corpuscular volume; MCH, mean corpuscular hemoglobin; MCHC, mean corpuscular hemoglobin concentration; RDW, red cell distribution width

Complete blood cell panel	Initial labs on the first admission (Day 1)	Labs on the first discharge (Day 6)	Initial labs on the second admission (Day 9)	Labs on the second discharge (Day 18)	Reference range and units
WBC	9.3	9.2	8.7	13.8	3.6-11.2 K/uL
RBC	2.69	3.73	2.72	2.82	3.63-4.92 M/uL
Hemoglobin	7.0	10.1	7.3	7.6	11.4-15.0 g/dl
Hematocrit	20	30	21	23.9	31-42%
MCV	76	80	79	85	74-96 fL
MCH	26	27	27	26.9	26-33 pg
MCHC	34	33	34	31.6	33-36 g/dL
RDW	14.3	15.2	15.8	17.8	12.3-17.0%
Platelets	8	242	11	376	150-450 K/uL
Percent reticulocytes	2.8	N/A	2.6%	N/A	0.5-2.2%

In the emergency room, the patient's vital signs were normal. Physical examination revealed conjunctival pallor and mild abdominal tenderness on palpation. No rashes, purpura, or petechiae was present. The neurological exam was unremarkable. The comprehensive metabolic panel (CMP) was only significant for an elevated total bilirubin level of 2.1 mg/dL. Hemolysis labs were obtained, and the patient was found to have a haptoglobin level of less than 30 mg/dL, lactate dehydrogenase (LDH) of 643 U/L, fibrinogen of 389 mg/dL, D-dimer of 3,583 ng/mL, and a reticulocyte count of 2.8% (Table 2). Normocytic anemia and thrombocytopenia with schistocytes were found on the peripheral blood smear. Ultrasound revealed a live twin intrauterine pregnancy.

**Table 2:** Hemolysis labs

Hemolysis labs	First admission	Second admission	Reference range and units
Haptoglobin	<30	<30	44-315 mg/dL
Bilirubin	2.1	1.9	0.3-1.0 mg/dL
Fibrinogen	389	370	200-500 mg/dL
D-dimer	3,583	1,026	<231 ng/ml DDU
Percent reticulocytes	2.8%	2.6%	0.5-2.2%
Schistocytes	Present	10-13 per HPF	None present

Hematology and nephrology were consulted, and the differential diagnosis considered for the patient's condition included several possibilities, given the complex clinical presentation. These included thrombotic thrombocytopenic purpura (TTP), immune thrombocytopenic purpura (ITP), systemic lupus erythematosus (SLE), preeclampsia, vasculitis, and anti-phospholipid syndrome, as well as autoimmune hemolytic anemia.

Many of these possibilities were systematically ruled out through a combination of clinical and laboratory assessments. For instance, the patient's blood pressure remained within normal limits, effectively eliminating preeclampsia as the primary cause of her symptoms. Furthermore, specific tests for anti-cardiolipin antibodies, beta-2 glycoprotein, and lupus anticoagulant all returned negative, thus decreasing the likelihood of an underlying anti-phospholipid syndrome.

The patient's rheumatological laboratory tests revealed the presence of positive anti-Sjögren's-syndrome-related antigen A (SSA) antibodies and a positive antinuclear antibody (ANA) test at a titer of 1:160, displaying a speckled pattern. However, it is crucial to consider that these tests were conducted after plasmapheresis (PLEX), which can introduce complexity when interpreting these serological findings. Plasmapheresis is known to produce results that can be both falsely positive and falsely negative in autoimmune serologies, potentially complicating the clinical assessment. Importantly, the patient did not exhibit any clinical symptoms strongly indicative of an underlying systemic rheumatic disease. 

The presence of schistocytes in the blood smear, a classic finding of microangiopathic hemolytic anemia, strongly suggested the diagnosis of TTP. Other supportive evidence included nonimmune hemolytic anemia (negative Coombs) and a high plasmic score of seven, which suggests a 72% risk of severe ADAMTS13 deficiency. With this compelling evidence, a high level of suspicion for TTP was established, leading to the immediate initiation of methylprednisolone (1 mg/kg) and plasmapheresis treatments.

The patient received a unit of packed red blood cells (PRBCs), three plasma exchange treatments, and scheduled methylprednisolone every eight hours over the course of three days and had an excellent response. The platelet count after three days was 242 x 10^3^/μL, and the hemoglobin level was stable at 10 g/dL (Table 1). The patient was discharged safely with obstetrics and hematology follow-up in three days.

Three days later, the patient was on her way to the hematology office when she developed a sudden onset headache and shortness of breath with exertion. Upon arrival to the emergency room, she was found to be anemic, with a hemoglobin level of 7.3 g/dL and a hematocrit level of 21%. In addition, there was a significant drop in her platelet count, which measured at 11 x 10^3^/μL (Table 1). A repeat peripheral smear indicated the presence of anemia, thrombocytopenia, and schistocytes (10 to 13 per high-power field). Hemolysis labs also returned positive results. At this point, no other potential causes were considered, and the patient's treatment for rTTP was initiated with plasmapheresis and prednisone (1 mg/kg), with the addition of caplacizumab (11 mg IV 15 minutes prior to plasmapheresis and 11 mg subcutaneously daily).

This time, after three plasmapheresis treatments, the platelet counts only increased to 24 x 10^3^/μL and plasmapheresis was continued. The OBGYN team had concerns that the patient's pregnancy might be exacerbating her TTP due to the prothrombotic nature of the pregnancy. While they could not provide absolute certainty, they held the belief that continuing the pregnancy would elevate her chances of experiencing another TTP episode. After discussing the risks with the patient and her family, the decision was made to terminate the pregnancy with a D&C procedure once the patient's platelet count was above 50,000/μL. Rituximab at 375 mg/m^2^ once weekly was also initiated when the ADAMTS13 level returned and was low at 2%. Plasmapheresis was continued for a total of 10 treatments on the second admission. The patient was taken to the operating room and a fetal ultrasound was done, which showed no cardiac activity in the fetal poles. A D&C was performed for a missed abortion. She was subsequently discharged three days later with a platelet count of 376 x 10^3^/μL (Table 1), a three-week prednisone taper (starting with 60 mg and decreasing by 20 mg every five days), daily caplacizumab (11 mg daily for 30 days), and weekly rituximab (375 mg/m^2^ once weekly for four doses). The patient was doing well on the day of discharge and had a scheduled close follow-up with OBGYN and hematology.

## Discussion

TTP is a primary TMA caused by severe ADAMTS13 deficiency either due to an inhibitor autoantibody or a biallelic mutation in the ADAMTS13 gene. ADAMTS13 is a protease that cleaves VWF molecules into smaller-sized multimers preventing large multimers from accumulating in high shear stress. When a deficiency is present, the VWF multimers accumulate in microvessels leading to the formation of platelet thrombi. This pathway causes microangiopathic hemolytic anemia and consumption thrombocytopenia, both of which are the hallmarks of TTP. Many patients may not have signs or symptoms until a triggering exposure, such as an infection or pregnancy. Pregnancy is a common precipitating event for TTP due to the hypercoagulable state, decreased activity of ADAMTS-13, and fibrinolysis. Because TTP can present in so many different ways during pregnancy, its phenotype and management are not well understood.

The majority of TTP pregnancy cases occur in the second and third trimesters. First-trimester-related TTP cases are rare, and therapeutic abortion is often recommended to prevent a recurrence [1]. It can also be challenging to differentiate between TTP in pregnancy versus HELLP (hemolysis, elevated liver enzymes and low platelets) syndrome, which can result in a delay in diagnosis [2]. Luckily, our patient did not have elevated liver enzymes, but given her history of preeclampsia, this remained on the differential. 

Initial symptoms of TTP include fatigue, dyspnea, petechiae, or other bleeding manifestations. Bleeding typically occurs due to the combination of thrombocytopenia, vascular injury, and/or tissue infarction. Some patients are asymptomatic and only diagnosed when complete blood count (CBC) reveals thrombocytopenia. The clinical pentad of fever, anemia (hemoglobin less than 10), thrombocytopenia (platelets less than 30,000), renal dysfunction, and neurological deficits is only present in less than 5% of TTP cases [3]. Renal dysfunction can present as decreased kidney function, acute kidney injury (AKI), or proteinuria on urinalysis. Neurological involvement can manifest as headache, confusion, focal deficits such as difficulty speaking, or transient numbness or weakness and may progress to seizure or coma in severe cases. 

The diagnosis of TTP is made based on clinical presentation and lab tests that show evidence of decreased platelets, red blood cells, and hemolysis. ADAMTS13 is not an immediate lab result and may take a few days to result. The use of the PLASMIC (platelet count, hemolysis labs, absence of active cancer, absence of stem-cell or solid-organ transplant, MCV, INR, creatinine) score is used to predict the likelihood of ADAMTS13 activity. A score of six to seven points is a high probability of TTP, and a score of zero to four points is a low probability of TTP [4]. 

With a high plasmic score, our patient was emergently started on plasma exchange therapy (PLEX). Plasma exchange is the mainstay of treatment for TTP. If untreated, TTP can lead to organ failure and death. With the advancement of PLEX, mortality rates have decreased from 90% to 10% among patients with acquired TTP. Along with plasma exchange, glucocorticoids are also started in the initial treatment for patients with high-risk features. PEX is continued until platelets are greater than 150,000. Steroids are continued until the ADAMTS13 level is above 20-30% and then tapered over two to four weeks [5]. 

Of the patients with an acute acquired TTP episode, 20% will not respond to treatment or will respond transiently, but then experience relapse within a few days [6]. This presentation is consistent with primary rTTP in which an alternative treatment is initiated. 

Caplacizumab is a humanized monoclonal antibody that is used to treat resistant/rTTP. This antibody blocks vWF interactions with platelet GPIb-IX-V, reducing the formation of microthrombi. Caplacizumab is a novel treatment for TTP that has shown efficacy in both acquired and congenital TTP in patients who have not responded to standard treatments [7,8]. It is a humanized bivalent variable domain immunoglobulin that targets the A1 domain of vWF, which plays a key role in the formation of platelet thrombi in TTP. By inhibiting the binding of vWF to platelets, caplacizumab reduces the formation of platelet-rich thrombi, thereby inhibiting microvascular thrombosis and preventing the progression of TTP [8,9]. It has been approved by the US Food and Drug Administration (FDA) and European Medicines Agency (EMA) for the treatment of acquired TTP in adults. The HERCULES trial, a phase three multicenter, randomized, double-blind study, demonstrated the safety and efficacy of caplacizumab in patients with acquired TTP. In the HERCULES trial, 145 patients with TTP were randomly assigned to receive caplacizumab (10 mg IV loading bolus, followed by 10 mg daily subcutaneously) or a placebo during plasma exchange and for 30 days thereafter. The primary outcome of the trial was the time it took for the platelet count to normalize. The median time to normalization of the platelet count was shorter in the caplacizumab group (2.69 days) than in the placebo group (2.88 days). The secondary outcomes of the trial included the risk of death, recurrence of TTP, and thromboembolic events. The risk of these events was lower in the caplacizumab group than in the placebo group. For example, the percentage of patients who had a composite outcome event (death, recurrence of TTP, or a thromboembolic event) was 74% lower in the caplacizumab group than in the placebo group. 

The results of the HERCULES trial showed that caplacizumab is an effective treatment for TTP. Caplacizumab is associated with a shorter time to normalization of the platelet count and a lower risk of death, recurrence of TTP, and thromboembolic events [10]. 

In pregnant women with TTP, caplacizumab has been used successfully as an adjunct to plasmapheresis and steroids, to achieve remission of the disease and decrease the risk of TTP-related complications [11]. According to the case report, caplacizumab was transferred across the placenta and detected in amniotic fluid and fetal blood in a patient with TTP. The estimated concentration in maternal blood was higher than in fetal blood, but it remains unclear if the dose was insufficient to prevent fetal thrombosis or if thrombosis was caused by pre-caplacizumab microthrombi formation. The authors concluded that caplacizumab treatment was safe and effective for the mother, but it may have been applied too late to save the fetus. They further suggest that early and determined initiation of caplacizumab treatment in TTP cases in early pregnancy, along with glucocorticoids and plasma exchange, may be reasonable to prevent thrombotic microangiopathy and fetal loss, despite the potential risk of pregnancy-specific hemorrhagic complications. 

Caplacizumab is a promising treatment for TTP, including refractory cases, and has shown effectiveness in pregnant women. However, there is a lack of large-scale studies that evaluate its safety and efficacy in pregnant patients with TTP. It is crucial to note that caplacizumab poses potential risks of fetal harm, so it should be used with caution in pregnant patients with TTP. Therefore, it is recommended to carefully consider the risks and benefits of caplacizumab in each individual case, through shared decision-making and after a thorough risk-benefit discussion. Further research is necessary to investigate its long-term safety and efficacy in this patient population. The American Society of Hematology guidelines recommend the use of caplacizumab in combination with plasma exchange and immunosuppressive therapy in patients with acquired TTP [12]. 

Rituximab is a monoclonal antibody that targets the CD20 antigens expressed on B cells and has been shown to be effective in the treatment of rTTP. Several case reports and small case series have demonstrated the efficacy of rituximab in patients with rTTP, either as a monotherapy or in combination with PEX [13]. Rituximab works by eliminating the pathogenic B cells that produce the autoantibodies against ADAMTS13 and can restore ADAMTS13 activity, leading to the remission of TTP [13]. 

The use of rituximab in pregnant women with rTTP is challenging due to the potential adverse effects on fetal development. There is limited data on the use of rituximab in pregnant women with TTP, and most of the published studies are case reports or small case series. A retrospective study by Fakhouri et al. [14] analyzed the outcomes of 13 pregnancies in 11 women with TTP who received rituximab during pregnancy. The study showed that the rituximab treatment did not result in any major fetal malformations, but there were three cases of neonatal thrombocytopenia. The authors concluded that rituximab can be considered a safe option for pregnant women with TTP who do not respond to PEX or who have frequent relapses. On the contrary, the authors emphasized the importance of first trying other treatment options and the delivery of the fetus before using immunosuppressive therapies, including rituximab [14]. 

According to a study by Scully et al. [15], available data suggest that rituximab may be a safe and effective treatment option for pregnant women with TTP. The study reported successful outcomes in pregnant women who received rituximab for TTP during the second or third trimester. One case report described a pregnant woman who received rituximab in her second trimester and showed a rapid response with improvement in platelet count, hemoglobin, and lactate dehydrogenase levels. She delivered a healthy newborn at term with no signs of rituximab-related adverse effects. Similarly, a case series of five pregnant women with TTP who received rituximab during pregnancy all showed improvement in platelet count and resolution of TTP symptoms, with four of the five women delivering healthy infants with no signs of rituximab-related adverse effects [15]. 

In conclusion, rituximab appears to be a viable treatment option for pregnant women with rTTP who are unresponsive to PEX or experience frequent relapses. While limited, the available data suggest that rituximab is both safe and effective for maternal and fetal outcomes. However, the potential risks of fetal malformations and neonatal thrombocytopenia should be carefully weighed against the potential benefits of the treatment. Close monitoring of both the mother and fetus is essential throughout rituximab treatment in pregnant women with rTTP. 

In our case report, caplacizumab and rituximab were used as salvage therapy for rTTP in a pregnant woman who failed to respond to plasmapheresis and steroids. Despite the treatment, the patient had a poor fetal outcome, which highlights the importance of early diagnosis and prompt initiation of therapy for TTP in pregnancy.

## Conclusions

TTP is a rare but serious complication of pregnancy that can present in the first trimester. In this particular case, pregnancy may have been a contributing risk factor. Our case highlights the challenges faced in managing rTTP in first-trimester pregnancy and the effects it can have on the fetus, the mother, and the outcome of the pregnancy.

For our patient, who has a confirmed history of rTTP, it is crucial that, in the event of pregnancy, she receives close monitoring from maternal fetal medicine due to her high-risk status. Prompt recognition and treatment are crucial for a good outcome. Timely delivery and appropriate management during labor and delivery play a pivotal role in ensuring the best possible outcome for both the mother and the baby.

Plasmapheresis remains the mainstay of treatment, but adjunctive therapies, such as caplacizumab and rituximab, are necessary for refractory cases. It is important to underscore that TTP is a complex and potentially life-threatening condition that demands a multidisciplinary approach involving obstetrics, hematology, and critical care for diagnosis, treatment, and management. The success of the treatment plan depends on the patient's unique circumstances and the underlying causes of their TTP.
